# Barriers, facilitators and implementation strategies to implement ‘patient’s own medication’ and ‘self-administration of medication’ in hospitals

**DOI:** 10.1093/intqhc/mzaf038

**Published:** 2025-04-28

**Authors:** Elisabeth M Smale, Jessica van den Berg, Jennifer Korporaal - Heijman, Charlotte L Bekker, Bart J F van den Bemt

**Affiliations:** Department of Pharmacy, Radboud University Medical Center, Geert Grooteplein Zuid, Nijmegen, Gelderland 6525 GA, The Netherlands; Department of Adult Intensive Care, Erasmus Medical Centre, Dr. Molewaterplein 40, Rotterdam, Zuid-Holland 3015 G, The Netherlands; Department of Pharmacy, Radboud University Medical Center, Geert Grooteplein Zuid, Nijmegen, Gelderland 6525 GA, The Netherlands; Department of Pharmacy, Frisius Medical Centre, Thialfweg 44, Heerenveen, Friesland 8441 PW, The Netherlands; Department of Pharmacy, Radboud University Medical Center, Geert Grooteplein Zuid, Nijmegen, Gelderland 6525 GA, The Netherlands; Department of Pharmacy, Radboud University Medical Center, Geert Grooteplein Zuid, Nijmegen, Gelderland 6525 GA, The Netherlands; Department of Pharmacy, Sint Maartenskliniek, Hengstdal 3, Ubbergen, Gelderland 6574 NA, The Netherlands

**Keywords:** hospitalization, inpatients, medication management, self-administration of medication, implementation science, qualitative research

## Abstract

**Background:**

Implementing patient’s own medication (POM) and self-administration of medication (SAM) has several benefits for safe and sustainable medication use, including enhanced patient empowerment reduced workload for hospital staff and decreased medication waste. Despite positive attitude of stakeholders, the upscaling of these strategies in hospitals remained limited. This study aimed to (i) identify barriers and facilitators for implementing POM and SAM and (ii) develop implementation strategies to address these.

**Methods:**

Semistructured interviews were conducted among healthcare providers involved in the implementation of POM and SAM in 10 Dutch hospitals. The study population encompassed (hospital and outpatient) pharmacists, pharmacy technicians, nurses, and (ward) physicians. The topic guide was based on COM-B model. Barriers and facilitators were identified with thematic content analysis and were categorized to the Consolidated Framework for Implementation Research (CFIR). Implementation strategies were selected based on identified barriers through the CFIR- Expert Recommendations for Implementing Change (ERIC) tool and identified strategies were clustered into predefined focus areas to develop implementation targets.

**Results:**

The 23 participants generally expressed a positive attitude towards implementation of POM and SAM. Themes reflecting facilitators related to (i) multiple benefits for patients, hospital, and society, (ii) a dedicated multidisciplinary implementation team, (iii) an iterative implementation process, whereas barriers related to (iv) substantial and invasive workflow changes, (v) reluctance to change responsibilities of healthcare providers, and (vi) unclear regulations and reimbursement. The CFIR-ERIC tool highlighted 57 implementation strategies in nine key focus areas to support the implementation of POM and SAM.

**Conclusion:**

To implement POM and PAM successfully, strategies relating to involving stakeholders, changing infrastructure, and using an iterative implementation process are required.

## Introduction

Safe and sustainable medication uses are major challenges under the growing demands and constrained resources of current healthcare systems. With complex comorbidities and advancements in treatments, medication use is getting more advanced. Simultaneously, an ageing population requires more medical care, while staff shortages and financial constraints strain capacity. Healthcare’s environmental impact is substantial [1], accounting for 4% of global carbon emissions [2], rising to 7% in developed countries like the Netherlands [3]. Medications are environmental hotspots [3–7]. Sustainability is seen as an essential component of quality improvement [8, 9], requiring a revaluation of medication use practices to maintain safety, sustainability, and high-quality care.

Upon hospitalization, patients’ home medications are typically replaced by alternatives from the hospital’s formulary. Medications are switched back at discharge, and redundant medications are then disposed of. A more sustainable approach would be to use ‘patient’s own medication’ (POM) during hospitalization. POM requires less time of hospital staff, wastes less medication, and saves money [10], while it can benefit patients when combined with ‘self-administration of medication’ (SAM) [11]. SAM allows capable patients to store and administer their own medications with healthcare providers supporting them, improving patients’ knowledge and adherence [12] to prevent common medication errors like erroneously restarting medications that were stopped during hospitalization [13]. Taken together, implementing POM and SAM can enhance safety and sustainability of medication use both in hospitals and at home.

Implementing POM and SAM in Dutch hospitals has several benefits, including improved medication safety, reduced staff workload, costs, and waste [14]. Qualitative research identified prerequisites for implementing SAM at various levels [15]. However, despite a few pilot studies [16], for unclear reasons, POM and SAM have not been broadly implemented in hospitals to date. This study aimed to (i) identify barriers and facilitators for implementing POM and SAM and (ii) develop implementation strategies to address these.

## Methods

### Design and setting

This study employed a qualitative research design by conducting semi-structured interviews with healthcare providers involved in the implementation of POM and SAM in 10 different hospitals throughout the Netherlands (Supplement 1).

In the Dutch healthcare system, medications are typically prescribed by physicians. Pharmacists and pharmacy technicians are responsible for ensuring medication safety, dispensing medication, and patient counselling. Upon hospital admission, pharmacy technicians verify patients’ medications and replace patients’ home medications by alternatives from the hospital’s formulary. Nurses are responsible for administering medications to patients during hospitalization.

Although some differences in tasks and responsibilities between hospitals exist, the tasks and responsibilities of pharmacy technicians and nurses change upon implementation of POM and SAM. Pharmacy technicians verify the medication that patients bring from home and patient’s ability to self-manage their medications during an interview prior to hospital admission. Nurses are now responsible for supervising medication use. In case of (temporary) incompetence to SAM, nurses can change the medication policy. Ward physicians remain responsible for overseeing treatment plans and making clinical decisions.

Given the medication-related objectives of POM and SAM, it was mostly initiated by the medication committee and/or pharmacy. Consequently, in most hospitals, the pharmacy was in the lead for implementation (in close collaboration with the management of the ward) and collaborated with physicians and nurses employed by the specific wards of interest. In a few cases, an external project leader or quality assurance officer was appointed to lead implementation of SAM.

### Ethical and administrative aspects

The study adheres to the Declaration of Helsinki, with all participants providing oral informed consent. Ethical approval was waived by the Radboud University Medical Centre’s ethics committee (protocol 2024-17 274). The study followed the Consolidated Criteria for Reporting Qualitative Research (COREQ) guidelines for comprehensive reporting (Supplement 2).

### Study population

The study population were healthcare providers involved in the implementation of POM and SAM in a Dutch hospital, including (hospital and outpatient) pharmacists, pharmacy technicians, nurses, and (ward) physicians.

A maximum variation sample was composed based on the hospital of employment (e.g. geographic location, type, size, and implementation status), and the professional role of the interviewee [17]. Purposive sampling was used to select a sample of nine healthcare providers responsible for implementation of POM and SAM (e.g. project leaders) in different hospitals [17]. Based on the outcomes of these interviews, a second sample of involved healthcare providers was selected in hospitals with differences in implementation successes using snowball sampling techniques. Recruitment was performed personally by e-mailing the participants with information about the interview and its purpose. After 2 weeks a reminder was sent to schedule an interview.

Data saturation was defined as new data being merely a repletion of predefined codes and themes [18], which was determined by two independent researchers (J.B. and E.S.) for each subgroup of healthcare providers and verified in two more interviews.

### Interviews

The topic guide was developed based on the COM-B model of behavioural change [19], which describes behaviour as the consequence of capability (C), opportunity (O), and motivation (M) (Supplement 3). The topic guide was pilot-tested in the first two interviews, and regularly evaluated in bi-weekly meeting with the research team; no questions were added.

The interviews were conducted by four interviewers; no other individuals were present during the interviews besides the participants and interviewers. The initial interviews with project leaders were conducted collaboratively by three independent interviewers, who were experienced with interviewing but new to the field of pharmacy, and had the assignment of developing implementation materials for POM and SAM (see ‘Acknowledgements’). The additional interviews were conducted by a fourth interviewer (J.B.), who was a bachelor student in pharmacy and was trained during the prior interviews. The pilot interviews were conducted and subsequently evaluated with a postdoctoral researcher in clinical pharmacy, experienced in qualitative research (E.S.), to refine the interview process and enhance the overall quality and consistency of the data collection. The interviews were conducted in a private meeting room at the site of employment of the interviewee or digitally through Microsoft Teams (version 4.2.4.0), depending on the preference of the interviewee.

The interviews were recorded. Verbal consent was obtained prior to the interviews. Interview recordings were transcribed verbatim using Good Tape Transcription—Confidential AI Transcription [20] and were then corrected for linguistic mistakes and anonymized by the interviewer. Upon completion of the data collection, healthcare providers were given the opportunity to verify the findings through a written survey through all interviewees, but no modifications have been made.

### Data analysis

With thematic content analysis, barriers and facilitators were identified [21]. Two interviewers first open-coded transcripts, with the first five interviews coded collaboratively with the first author (E.S.). All codes were then checked for consistency, resolving differences by consensus. Axial codes were developed by the second author (J.B.) and discussed with E.S. until consensus was reached. Finally, J.B. and E.S. discussed interrelationships among the axial codes to identify themes reflecting barriers and facilitators. These were categorized into the updated Consolidated Framework for Implementation Research (CFIR) [22], which describes factors influencing implementation by 48 constructs categorized in five domains: (i) innovation, (ii) outer setting, (iii) inner setting, (iv) individuals, and (v) implementation process. The Expert Recommendations for Implementing Change (ERIC) provide 73 implementation strategies [23]. Using the CFIR–ERIC Implementation Strategy Matching tool, strategies were selected based on identified barriers, with output percentages reflecting expert consensus on effectiveness. Strategies were categorized into Level-1 (top strategies endorsed by ≥50% of experts) and Level-2 (endorsed by 20–50% of experts) [24]. These strategies were grouped into key focus areas based on previously defined clusters to guide the implementation of POM and SAM [25].

## Results

A total of 24 healthcare providers employed in 10 hospitals were approached for an interview, of whom 23 healthcare providers employed in 10 hospitals were interviewed (Table 1). The reasons for not participating was lack of response (*n* = 1). A majority of participants (*n* = 13) were interviewed at their site of employment, whereas 10 participants were interviewed throughout Microsoft Teams.

**Table 1.** Participants’ characteristics.

Participant(s)	Hospital	Hospital type	Implementation status*	Project leader	Occupation
P1	1	General	+	X	Hospital pharmacist
P2	2	Specialized	+ +	X	Hospital pharmacist
P3	3	Academic	±		Hospital pharmacist
P4	3	Academic	±		Nurse
P5	3	Academic	±		Nurse
P6	3	Academic	±		Hospital pharmacist
P7	3	Academic	±	X	Pharmacy technician
P8	3	Academic	±		Outpatient pharmacist
P9	3	Academic	±		Physician
P10	4	General	-	X	Hospital pharmacist
P11	5	Academic	-	X	Hospital pharmacist
P12	6	Teaching	+	X	Hospital pharmacist
P13	6	Teaching	+		Nurse
P14	6	Teaching	+		Pharmacy technician
P15	6	Teaching	+		Outpatient pharmacist
P16	6	Teaching	+		Physician assistant
P17	7	Teaching	-	X	Hospital pharmacist
P18	8	Teaching	+	X	Hospital pharmacist
P19	9	Teaching	- -		Hospital pharmacist
P20 + 21	9	Teaching	- -	X	Pharmacists
P22	10	Academic	-	X	Nurse
P23	10	Academic	-		Nurse

* Implementation status is defined as POM and SAM fully implemented through medical center (+ +), partially implemented (+), pilot-tested (±), not yet implemented (−) or implementation unsuccessful (− −).

Participants generally expressed a positive attitude towards implementation of POM and SAM. Themes reflecting barriers and facilitators to implementation (Table 2) encompassed all CFIR constructs. Facilitators related to (i) multiple benefits for patients, hospitals, and society; (ii) a dedicated project team; (iii) an iterative implementation process, whereas barriers related to (iv) substantial and invasive workflow changes; (v) reluctance to change responsibilities of healthcare providers; and (vi) unclear regulations and reimbursement, while per identified barrier or facilitator illustrative quotes are summarized in Table 3.

**Table 2.** Themes reflecting barriers and facilitators to implementing POM and SAM with corresponding CFIR constructs.

	Themes	Content	CFIR constructs
Barriers	Substantial and invasive workflow changes	All medication procedures must be changed for implementing POM and SAM, which is hindered by: Technological barriers Structural characteristics	**Innovation**—*Complexity* **Inner setting—** *Compatability* *Structural characterics*
	Reluctance to change responsibilities of healthcare providers	Implementing POM and SAM requires behavioral change of healthcare providers: Letting go of the responsibility of administrating medications Executing new tasks like educating patients	**Individuals**—*Capability*
	Unclear regulations and reimbursement	Absence of clear regulations raises concerns regarding: Liability Financial discrepancies	**Outer setting**—*Policies and laws*
Facilitators	Multiple benefits for patients, hospital, and society	POM and SAM can have multiple advantages, supporting implementation: Increasing safe use More patient empowerment Reduction of staff time Innovation & research Reducing medication waste	**Innovation**—*Relative Advantage*
	A dedicated multidisciplinary implementation team	A dedicated project team helping implementation of POM and SAM: Having a (high-authority) project leader Having an implementation team Involving nurses, physicians, and pharmacy technicians	**Individuals**— *Implementation Leads* *High-Level Leader* *Implementation Team Members* *Innovation deliverers*
	An iterative implementation process	An iterative process allows for gradual, well-informed implementation of POM and SAM: Planning Conducting a pilot-study Reflecting and evaluating Scaling-up	**Implementation**— *Planning* *Doing* *Reflecting and evaluating* **Innovation**—*Trialability*
		Several strategies supporting implementation were identified: Engagement of stakeholders Exchanging information and protocols Having a strategic planning Having a positive business case	**Implementation**—*Engaging* **Inner setting**— *Available resources* *Access to knowledge and information*

CFIR: The Updated Consolidated Framework for Implementation Research.

**Table 3.** Illustration of the participants’ quotes matching the identified barriers and facilitators.

Barrier/facilitator	Participant	Quote
Multiple benefits for patients, hospital, and society	P18	‘The driving forces were in particular to promote patient safety and decrease medication wastage. All medications that we dispose now just feels like a real waste. So we saw a lot of value in that [POM and SAM] and just guiding the patient better, providing more education. Those were kind of the two main points that we recognized and prioritized’.
A dedicated multidisciplinary implementation team	P3	‘I think it’s helpful anyway if there are some key figures, who have enough influence. Who can have enough persuasive power and influence to realize this. So I think that’s an important one. Yes, I myself have always acted that way I think. You’re also a teacher, a hospital pharmacist, you know everything about safety, so you have the authority to push …. Certainly at those times when incidents came up, that was very important I think.’
	P6	‘We had two project groups, one from the pharmacy and one from the nursing department .. so starting at the pharmacy, what options are there? What do we think is a convenient way of working? You then connect that to the nurses: “could this work for you guys?”. Usually it was “Um, yeah, we can think of something”, but if it’s not workable for the nurse, yeah, that’s not going to be successful. And vice versa. So you do have to find alignment on that, that’s how it works for us’.
Reluctance to change responsibilities of healthcare providers	P11	“For nurses it is quite difficult to let go of that [medication administration]. We always had to scan and register everything that was administered. That is an important cultural shift.”
	P1	‘Keeping it safe and having it clear whether somebody can still do it themselves. I think those are the two biggest bottlenecks. What I find most difficult to manage is when someone no longer has to take medication from home. To have that clear.’
Substantial and invasive workflow changes	P12	‘And the challenge lies mainly in that the outpatient pharmacy needs to know which prescriptions are POM/SAM prescriptions. And which department they need to go to and when. And that’s very difficult.’
An iterative implementation process	P6	‘Yes, in the beginning, we really went quite far to provide comfort to the nurses. They had rather bizarre demands. We couldn’t even fulfil all of them. We installed all sorts of things to calm them down, like a red bag to dispose of medication that should not be used at home.’
	P2	‘In the beginning we also excluded quite a few categories of patients, because the ward physician thought that all of them, for example patients using monitored dosing systems or weekly boxes, or patients who had a certain procedure that made them physically unable to do it, that sort of things. But over time, we reviewed that and asked “is it really necessary to be so rigorous”. So step by step people could get used to it.’
Unclear regulations and reimbursement	P5	‘This is what many hospitals encounter: they are afraid of committing an economic offense.’
	P19	‘I think that what nurses fear most of all, is the legal responsibility. For when things go wrong. And that’s why I think we also need to have a statement of KNMP (Royal Dutch Society of Pharmacy), NVZA (Dutch Association of Hospital Pharmacists), or whatever.’

### Multiple benefits for patients, hospitals, and society

The perception that POM and SAM contributed to multiple purposes relating to patients, hospitals, and society was seen as a facilitator to implementation. Perceived benefits of SAM included increased medication safety and patient empowerment as SAM could optimize the transition from the hospital to patients’ home setting, potentially resulting in fewer medication errors. Furthermore, it was argued that patient involvement is becoming more ubiquitous, which should also be applied to medication, specifically since most patients preferred SAM.

On the hospital level, POM and SAM were associated with less nursing time. This was perceived beneficial due to the staff deficits. Additionally, a few participants perceived POM and SAM a means to distinguish themselves as an innovative hospital.

Since POM eliminates the need for duplicate medication supplies, it could address medication waste. This could result in lower medication costs and environmental impacts. It was therefore seen as a sustainability solution, benefiting society.

### A dedicated multidisciplinary implementation team

Changing medication processes for POM and SAM affects a broad range of healthcare providers, according to participants. Accordingly, a dedicated multidisciplinary implementation team was perceived a facilitator to implementation.

All participants recognized the importance of an implementation leader, who can oversee project planning and organization, manages questions, and is responsible for resolving obstacles. It was considered beneficial if filled by someone with authority, ensuring they could effectively oversee the process and create an encouraging collaboration within the hospital.

Participants also emphasized the need for a implementation team with representatives from the pharmacy and clinical ward (i.e. nurses and physicians) that can answer questions of colleagues and help to identify practical issues. They also perceived POM and SAM as interdisciplinary collaboration, requiring the knowledge of both disciplines for contextual understanding and, with that, effective change management.

### An iterative implementation process

To foster support and manage resistance, an iterative implementation process focussing on stakeholder engagement was seen as facilitator by participants. Structured procedures such as regular presentations, newsletters, and evaluation of pilot studies were employed, while clear communication of benefits was ensured.

For smooth integration in clinical practice, clear procedures are required. Participants emphasized the importance of exchanging knowledge, protocols, and implementation tools, such as information leaflets, with other medical centres, offering insights and solutions that could be adapted to the local context. Drafting a business case and using strategic planning were seen as facilitators.

Pilot-testing the procedures in selected wards with planned admissions was perceived helpful to identify and address barriers. It also helped to gain trust, so guidelines could be looser, for instance related to the inclusion and exclusion criteria for patients.

Continuous feedback and regular evaluations optimized the process and helped to address barriers, according to participants. One concern related to the time investment of nurses. Although SAM saves time for nurses, it requires time to teach patients how to administer their own medication. Similarly, POM may decrease intramural waste, but a few participants declared that waste in the home setting could increase. Accordingly, participants perceived a need for follow-up research, especially after patient discharge.

### Substantial and invasive workflow changes

POM and SAM aim to improve patient safety, making safe workflows a key challenge when reorganizing medication processes. Risks mentioned by participants included technological challenges, concerns of misuse by other patients and patients’ capability, and it was advised to conduct a local risk analysis prior to implementation.

Successful POM and SAM implementation requires coordination across multiple care settings hindered by electronic health records (EHR). Current systems do not allow nurses to order medications from outpatient pharmacies, leading to a labour-intensive process that can delay medication delivery according to participants. It would also require a structural change, for instance lockers at the bedside.

Mitigation strategies that were identified for the comprehensive change of medication-related processes included ongoing infrastructure changes and digital tools, such as tablets for self-registration.

### Reluctance to change responsibilities of healthcare providers

Participants found the shift of tasks and responsibilities associated with POM and SAM challenging. For instance, not registering medication administration, which is controlled through barcode scanning in some hospitals, caused uncertainties regarding accountability of nurses according to some participants. Another concern was incorrect medication use, requiring an adequate suitability assessment and proper education by nurses and pharmacy technicians. The use of supportive materials, such as instructional videos, mitigated concerns and made this transition less challenging to participants.

### Unclear regulations and reimbursement

Participants highlighted challenges due to the absence of clear regulations on liability and reimbursement structures, emphasizing the need for clearly defined responsibilities and liability in case of medication errors.

Absence of a reimbursement framework also posed an obstacle as it was argued that POM could potentially cause financial discrepancies due to the double reimbursement of medications [e.g. through diagnosis-related groups (DRCs) and to (outpatient) pharmacies]. While healthcare insurances have currently tolerated this on limited scale, participants indicated that formal financial agreements would be needed to support hospital-wide implementation.

### Implementation strategies for POM and SAM

In total, 32 Level-1 strategies and 25 Level-2 strategies were identified by the CFIR-ERIC tool (Supplement 4). Several strategies cut across multiple (≥4) barriers, including identify and prepare champions, promote adaptability, conduct cyclical small tests of change, assess for readiness by identifying barriers and facilitators, and capture and share local knowledge.

ERIC strategies corresponding to barriers of implementing POM and SAM were clustered into nine focus areas (Table 4): (i) develop stakeholder interrelationships; (ii) train and educating stakeholders; (iii) use evaluative and iterative strategies; (iv) use financial strategies; (v) provide interactive assistance; (vi) adapt and tailoring to context; (vii) change infrastructure; (viii) support clinicians; (ix) engage consumers.

**Table 4.** Focus areas of implementation strategies for POM and SAM.

Focus areas	Implementation strategies based on CFIR-ERIC tool	Expert agreement (Cumulative %)	Level of addressing CFIR-constructs
**Develop stakeholder interrelationships**	Identify and prepare champions	185	1
	Capture and share local knowledge	143	1
	Model and simulate change	124	1
	Identify early adopters	118	1
	Build a coalition	113	1
	Conduct local consensus discussions	112	1
	Inform local opinion leaders	107	1
	Organize clinician implementation team meetings	78	1
	Involve executive boards	66	1
	Use an implementation adviser	63	1
	Visit other sites	60	1
	Use advisory boards and workgroups	42	2
	Promote network weaving	42	2
	Develop academic partnerships	39	2
	Recruit, designate and train for leadership	33	2
	Obtain formal commitments	28	1
**Train and educate stakeholders**	Create a learning collaborative	149	1
	Conduct ongoing training	109	1
	Provide ongoing consultation	109	1
	Make training dynamic	99	1
	Conduct educational meetings	86	1
	Conduct educational outreach visits	61	1
	Shadow other experts	60	1
	Develop educational materials	59	1
	Use train the trainer strategies	37	2
	Distribute educational materials	27	2
**Use evaluative and iterative strategies**	Conduct cyclical small tests of change	173	1
	Assess for readiness and identify barriers and facilitators	162	1
	Stage implementation scale up	107	1
	Develop a formal implementation blueprint	107	1
	Purposely reexamine the implementation	75	1
	Conduct local needs assessment	69	1
	Audit and provide feedback	60	1
	Develop and implement tools for quality monitoring	49	1
	Develop and organize quality monitoring systems	48	1
	Obtain and use patients/consumers and family feedback	30	1
**Utilize financial strategies**	Alter incentive/allowance structures	112	1
	Fund and contract for clinical innovation	66	1
	Place innovation on fee for service lists/formularies	30	2
	Access new funding	23	2
**Provide interactive assistance**	Facilitation	110	1
	Provide local technical assistance	110	1
	Centralize technical assistance	40	2
	Provide clinical supervision	36	2
**Adapt and tailor to context**	Promote adaptability	174	1
	Tailor strategies	136	1
	Use data experts	22	2
**Change infrastructure**	Mandate change	49	2
	Change physical structure and equipment	42	2
	Create or change credentialing and/or licensure standards	31	2
	Change liability laws	25	2
	Change accreditation or membership requirements	23	2
	Change service sites	21	2
**Support clinicians**	Revise professional roles	36	2
	Facilitate relay of clinical data to providers	30	2
	Create new clinical teams	27	2
**Engage consumers**	Involve patients/consumers and family members	46	2

Supplement 3 describes the correlation of the implementation strategies to specific CFIR constructs.

## Discussion

### Statement of principal findings

This study identified barriers and facilitators to implementation of POM and SAM. Facilitators related to (i) benefits for patients, hospitals, and society; (ii) a dedicated multidisciplinary implementation team; and (iii) an iterative implementation process, whereas barriers related to (iv) substantial and invasive workflow changes; (v) reluctance to change responsibilities of healthcare providers; and (vi) unclear regulations and reimbursement. The CFIR–ERIC tool revealed nine focus areas for implementation strategies for POM and SAM, mainly relating to involving stakeholders, changing infrastructure, and using an iterative implementation process.

### Strengths and limitations

This study’s strength lies in its guidance for implementing POM and SAM by using the systematic, reproducible CFIR-ERIC tool for selecting strategies based on the experiences from involved healthcare providers. A diverse sample reflecting both successful and unsuccessful experiences provided a valid foundation for an implementation plan for POM and SAM. The study was, however, also prone to limitations, like its generalizability as barriers and facilitators related to the Dutch context. Extrapolation to other settings must therefore be done carefully, taken into account differences in roles and responsibilities of healthcare providers in global healthcare services. Another limitation of this study is that multiple individuals with different backgrounds conducted the interviews, which may have introduced variability due to differences in responses, interpretation and the mode-of the interviewing, potentially affecting the consistency of the data. Moreover, the CFIR–ERIC tool supports selection of potential implementation strategies, but does not necessarily provide guidance on how to adapt strategies to local contexts. Therefore, it is recommended to complement the current findings with a context analysis to select a final set of tailored implementation strategies. This can be achieved, for instance, through the use of the Basel Approach for coNtextual ANAlysis (BANANA) approach [26].

### Interpretation within the context of the wider literature

Identified barriers and facilitators to implementation of POM and SAM were mostly in line with previous studies. Specifically, a safe workflow and well-defined responsibilities corresponded to previously qualitative research regarding prerequisites for implementation of POM and SAM among patients and stakeholders [12, 15, 27, 28]. Also benefits for sustainability have been identified as a motivator for implementing POM before [29], which was verified with empirical data in a Dutch [10] and UK context [30].

Not all perceptions matched empirical evidence to date. Studies have not yet conclusively evaluated benefits of patient safety and adherence after implementing SAM [16, 31–33], while these benefits appeared key facilitators in this study. Similarly, the lack of reimbursement frameworks for POM was perceived as barrier, but a recent evaluation indicated that the minimal impact of reimbursement should not affect implementation [34]. It is therefore essential to validate perceptions with data and adjust implementation strategies accordingly, including regular feedback loops as highlighted in ‘iterative implementation process’.

### Implications for policy, practice, and research

**Figure 1:**
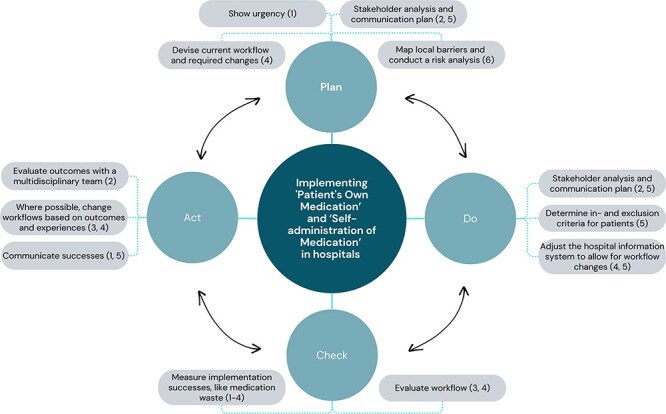
Implementation plan for ‘POM’ and ‘SAM’ in hospitals based on the barriers, facilitators, and implementation strategies identified in this study:

Interviews revealed inconsistencies in implementing POM and SAM, underscoring the need for uniform guidelines. Some issues related to practices, like the use of a certain distribution or IT systems, which favoured one approach over another. Others were due to incomplete implementation, like applying POM and SAM only to multidose medications (e.g. inhalers, dermatics) for convenience. A Belgian study showed that fewer than 20% of hospital wards had established SAM procedures, with <10% using screening tools for assessing patients’ ability to self-administer [35]. While standardized protocols exist in the UK [36] and Belgium [37], they lack implementation guidance. Based on the findings of this study, a comprehensive toolkit was developed for Dutch healthcare providers, featuring detailed procedures (as well as alternative options), protocols, frequently asked questions, and additional resources such as reimbursement information and patient materials, including instructional videos. This toolkit also encompasses an implementation plan, based on the barriers, facilitators, and implementation strategies identified in this study, to support teams to start with successfully implementing POM and SAM (Fig. 1).

(1) benefits for patients, hospitals, and society; (2) a dedicated multidisciplinary implementation team; and (3) an iterative implementation process, whereas barriers related to (4) substantial and invasive workflow changes; (5) reluctance to change responsibilities of healthcare providers; and (6) unclear regulations and reimbursement

## Conclusions

Several barriers and facilitators influenced the implementation of POM and SAM. Facilitators related to multiple benefits for patients, hospitals and society, a dedicated multidisciplinary implementation team, and an iterative implementation process. Barriers related to substantial and invasive workflow changes, reluctance to change responsibilities of healthcare providers and unclear regulations and reimbursement. Nine key areas for implementation strategies were identified. Implementation of POM and SAM should involve stakeholders, change infrastructure and use an iterative implementation process to be successful.

## Supplementary Material

mzaf038_Supp

## Data Availability

Due to the confidentiality of the interviewees that provided the qualitative data underlying this study, data cannot be made publicly available. Data will be shared on reasonable request to the corresponding author.

## References

[1] Romanello M, McGushin A, Di Napoli C et al. The 2021 report of the Lancet countdown on health and climate change: code red for a healthy future. *Lancet* 2021;398:1619–62. doi: 10.1016/S0140-6736(21)01787-6 34687662 PMC7616807

[2] Pichler PP, Jaccard IS, Weisz U et al. International comparison of health care carbon footprints. *Environ Res Lett* 2019;14:064004. doi: 10.1088/1748-9326/ab19e1

[3] Steenmeijer MA, Rodrigues JFD, Zijp MC et al. The environmental impact of the Dutch health-care sector beyond climate change: an input-output analysis. *Lancet Planet Health* 2022;6:e949–57. doi: 10.1016/S2542-5196(22)00244-3 36495889

[4] Lenzen M, Malik A, Li M et al. The environmental footprint of health care: a global assessment. *Lancet Planet Health* 2020;4:e271–9. doi: 10.1016/S2542-5196(20)30121-2 32681898

[5] Lau I, Burdorf A, Hesseling S et al. The carbon footprint of a Dutch academic hospital—using a hybrid assessment method to identify driving activities and departments. *Front Public Health* 2024;12: 1380400 doi: 10.3389/fpubh.2024.1380400 PMC11151853 38841663

[6] Weisz U, Pichler -P-P, Jaccard IS et al. Carbon emission trends and sustainability options in Austrian health care. *Resour Conserv Recycl* 2020;160:104862. doi: 10.1016/j.resconrec.2020.104862

[7] Tennison I, Roschnik S, Ashby B et al. Health care’s response to climate change: a carbon footprint assessment of the NHS in England. *Lancet Planet Health* 2021;5:e84–e92. doi: 10.1016/S2542-5196(20)30271-0 33581070 PMC7887664

[8] Mortimer F, Isherwood J, Wilkinson A et al. Sustainability in quality improvement: redefining value. *Future Healthc J* 2018;5:88–93. doi: 10.7861/futurehosp.5-2-88 31098540 PMC6502556

[9] The Centre for Sustainable Healthcare . *Helping you to Use SusQI*. 2024. [cited 2024 26 November]; Available from: https://www.susqi.org/ (26 November 2024, date last accessed).

[10] van Herpen-meeuwissen LJM, van den Bemt BJF, Derijks HJ et al. Economic impact of patient’s own medication use during hospitalisation: a multicentre pre-post implementation study. *Int J Clin Pharm* 2019;41:1658–65. doi: 10.1007/s11096-019-00932-1 31705458

[11] Salmon K Self-administration of Medicines and the Re-use of Patients Own Drugs. *Foundation of nursing studies dissemination series* 2002;3.

[12] van Herpen-meeuwissen LJM, Bekker CL, Cornelissen N et al. Patients’ views on self-administration of medication during hospitalisation: a mixed-methods study. *Ther Adv Drug Saf* 2022;13:20420986221107804. doi: 10.1177/20420986221107804 PMC9340381 35923715

[13] Wijker E, de Bie-daliri S, van den Bemt PM et al. The prevalence of medication errors after hospital discharge in patients with a medication change [Dutch]. *Dutch Platf Pharm Res* 2024;9 a1779.

[14] Meeuwissen L Patient participation in medication management during hospitalisation: empower to sustain. 2023.

[15] van Herpen-meeuwissen LJM, Djodikromo MF, Maat B et al. Inpatients self-administration of medication: stakeholders’ views and prerequisites. *J Clin Nurs* 2023;32:2709–21. doi: 10.1111/jocn.16369 35596267

[16] van Herpen-meeuwissen LJM, van Onzenoort HA, van den Bemt PM et al. The effect of self-administration of medication during hospitalization on patient’s self-efficacy and medication adherence after discharge. *Patient Prefer Adherence* 2022;16:2683–93. doi: 10.2147/PPA.S375295 36196066 PMC9527028

[17] Suri H Purposeful sampling in qualitative research synthesis. *Qual Res J* 2011;11:63–75. doi: 10.3316/QRJ1102063

[18] Saunders B, Sim J, Kingstone T et al. Saturation in qualitative research: exploring its conceptualization and operationalization. *Qual Quantity* 2018;52:1893–907. doi: 10.1007/s11135-017-0574-8 PMC5993836 29937585

[19] Michie S, van Stralen MM, West R The behaviour change wheel: a new method for characterising and designing behaviour change interventions. *Implement Sci* 2011;6:42. doi: 10.1186/1748-5908-6-42 PMC3096582 21513547

[20] Goodtape . *Good Tape: fast, secure and accurate transcription for all your audio and video*. 2025. [cited 2025 10 February]; Available from: https://blog.goodtape.io/?gad_source=1&gclid=CjwKCAiA5Ka9BhB5EiwA1ZVtvP7J-d3phUIVhZCl76K4tLy2PTIjNevAFBAL94l9HvUIxCqhNHiJgBoCGHcQAvD_BwE (10 February 2025, date last accessed).

[21] Braun V, Clarke V Using thematic analysis in psychology. *Qual Res Psychol* 2006;3:77–101. doi: 10.1191/1478088706qp063oa

[22] Damschroder LJ, Reardon CM, Widerquist MAO et al. The updated consolidated framework for implementation research based on user feedback. *Implement Sci* 2022;17:75. doi: 10.1186/s13012-022-01245-0 PMC9617234 36309746

[23] Powell BJ et al. A refined compilation of implementation strategies: results from the Expert Recommendations for Implementing Change (ERIC) project. *Implement Sci* 2015;10:21. doi: 10.1186/s13012-015-0209-1 PMC4328074 25889199

[24] Waltz TJ, Powell BJ, Fernández ME et al. Choosing implementation strategies to address contextual barriers: diversity in recommendations and future directions. *Implement Sci* 2019;14:42. doi: 10.1186/s13012-019-0892-4 PMC6489173 31036028

[25] Waltz TJ, Powell BJ, Matthieu MM et al. Use of concept mapping to characterize relationships among implementation strategies and assess their feasibility and importance: results from the Expert Recommendations for Implementing Change (ERIC) study. *Implement Sci* 2015;10:109. doi: 10.1186/s13012-015-0295-0 PMC4527340 26249843

[26] Mielke J, Leppla L, Valenta S et al. Unraveling implementation context: the Basel Approach for coNtextual ANAlysis (BANANA) in implementation science and its application in the SMILe project. *Implement Sci Commun* 2022;3:102. doi: 10.1186/s43058-022-00354-7 PMC9526967 36183141

[27] Vanwesemael T, Boussery K, Manias E et al. Self‐management of medication during hospitalisation: healthcare providers’ and patients’ perspectives. *J Clin Nurs* 2018;27:753–68. doi: 10.1111/jocn.14084 28960641

[28] Manias E, Rixon S, Williams A et al. Barriers and enablers affecting patient engagement in managing medications within specialty hospital settings. *Health Expect* 2015;18:2787–98. doi: 10.1111/hex.12255 25186633 PMC5810626

[29] Lummis H, Sketris I, Veldhuyzen van Zanten S Systematic review of the use of patients’ own medications in acute care institutions. *J Clin Pharm Therapeutics* 2006;31:541–63. doi: 10.1111/j.1365-2710.2006.00773.x 17176360

[30] Erotocritou M, Choa G, Clark OI et al. A quality improvement initiative to increase the use of patients’ own drugs through the implementation of a ‘Green Bag’ scheme at a central London hospital. *Future Healthc J* 2019;6:31. doi: 10.7861/futurehosp.6-1-s31 PMC6616769 31363556

[31] Polito S, Ho L, Pang I et al. Evaluation of a patient self-medication program in allogeneic hematopoietic stem cell transplantation. *J Oncol Pharm Pract* 2022;28:1790–7. doi: 10.1177/10781552211043525 34569857 PMC9623336

[32] Beigloo RHA et al. Self-administered medications in cardiovascular ward: a study on patients’ self-efficacy, knowledge and satisfaction. *Evidence Based Care* 2019;9:16–25.

[33] Hajialibeigloo R, Mazlum SR, Mohajer S et al. Effect of self-administration of medication programme on cardiovascular inpatients’ medication adherence and nurses’ satisfaction: A randomized clinical trial. *Nurs Open* 2021;8:1947–57. doi: 10.1002/nop2.870 33811803 PMC8186674

[34] Verdijk JC et al. The effect of different delivery strategies on the level of double funding for patients continuing home medication during hospital admission [Dutch]. *Ned Platf Farm Onderz* 2024;9:a1777.

[35] Vanwesemael T, Van Rompaey B, Petrovic M et al. SelfMED: Self‐administration of medication in hospital: a prevalence study in Flanders. *J Nurs Scholarsh* 2017;49:277–85. doi: 10.1111/jnu.12290 28376562

[36] Medicines Committee NEL Self-administration of medicines (SAM) by inpatients. 2020.

[37] Vanwesemael T et al. An evidence-based procedure for self-management of medication in hospital: development and validation of the SelfMED procedure. *Pharmacy* 2018;6:77. 10.3390/pharmacy6030077 PMC6164845 30049965

