# Supplementary material for: Barriers, facilitators and implementation strategies to implement ‘patient’s own medication’ and ‘self-administration of medication’ in hospitals
Source: Int J Qual Health Care. 2025 Apr 28;37(2):mzaf038. doi: 10.1093/intqhc/mzaf038 (PMC12491946; doi:10.1093/intqhc/mzaf038)
Supplement: mzaf038_Supp [file mzaf038_supp.zip › suppl_data/20250304 Supplementary Data.docx]

# **Supplementary Data**

## **Supplement 1: List of participating medical center**

University medical centers

- University Medical Center Groningen located in Groningen
- Radboud university Medical Center located in Nijmegen
- Maastricht University Medical Center located in Maastricht

Teaching hospitals

- Jeroen Bosch Hospital located in ‘s Hertogenbosch
- Onze Lieve Vrouwe Gasthuis located in Amsterdam
- Elisabeth Tweesteden Hospital located in Tilburg
- Amphia Hospital located in Breda

Generic hospitals

- Bravis Hospital located in Bergen op Zoom and Roosendaal
- Sint Jansdal Hospital located in Hardewijk and Lelystad

Specialized hospital

- Sint Maartenskliniek located in Nijmegen

## **Supplement 2: COREQ Checklist applied to current study**

| **Topic** | **Item No.** | **Guide Questions/Description** | **Reported in (section)** |  |  |  |
| --- | --- | --- | --- | --- | --- | --- |
| **Domain 1: Research team and reflexivity** | | | |  |  |  |
| *Personal characteristics* |  |  |  |  |  |  |
| Interviewer/facilitator | 1 | Which author/s conducted the interview or focus group? | Interviews |  |  |  |
| Credentials | 2 | What were the researcher’s credentials? E.g. PhD, MD | Interviews |  |  |  |
| Occupation | 3 | What was their occupation at the time of the study? | Interviews |  |  |  |
| Gender | 4 | Was the researcher male or female? | Interviews |  |  |  |
| Experience and training | 5 | What experience or training did the researcher have? | Interviews |  |  |  |
|  | | | |  |  |  |
| Relationship established | 6 | Was a relationship established prior to study commencement? | Interviews |  |  |  |
| Participant knowledge of the interviewer | 7 | What did the participants know about the researcher? e.g. personal goals, reasons for doing the research | Interviews |  |  |  |
| Interviewer characteristics | 8 | What characteristics were reported about the interviewer/facilitator? e.g. Bias, assumptions, reasons and interests in the research topic | Interviews |  |  |  |
| **Domain 2: Study design** |  |  |  |  |  |  |
| *Theoretical framework* |  |  |  |  |  |  |
| Methodological orientation and Theory | 9 | What methodological orientation was stated to underpin the study? e.g. grounded theory, discourse analysis, ethnography, phenomenology, content analysis | Interviews, data analysis |  |  |  |
| *Participant selection* |  |  |  |  |  |  |
| Sampling | 10 | How were participants selected? e.g. purposive, convenience, consecutive, snowball | Study population |  |  |  |
| Method of approach | 11 | How were participants approached? e.g. face-to-face, telephone, mail, email | Study population |  |  |  |
| Sample size | 12 | How many participants were in the study? | Results |  |  |  |
| Non-participation | 13 | How many people refused to participate or dropped out? Reasons? | Results |  |  |  |
| *Setting* |  |  |  |  |  |  |
| Setting of data collection | 14 | Where was the data collected? e.g. home, clinic, workplace | Interviews, Results |  |  |  |
| Presence of nonparticipants | 15 | Was anyone else present besides the participants and researchers? | Interviews |  |  |  |
| Description of sample | 16 | What are the important characteristics of the sample? e.g. demographic data, date | Table 1 |  |  |  |

| **Topic** | **Item No.** | **Guide Questions/Description** | **Reported in (section)** |  |  |  |
| --- | --- | --- | --- | --- | --- | --- |
| *Data collection* | | | |  |  |  |
| Interview guide | 17 | Were questions, prompts, guides provided by the authors? Was it pilot tested? | Interviews |  |  |  |
| Repeat interviews | 18 | Were repeat interviews carried out? If yes, how many? | Interviews |  |  |  |
| Audio/visual recording | 19 | Did the research use audio or visual recording to collect the data? | Interviews |  |  |  |
| Field notes | 20 | Were field notes made during and/or after the interview or focus group? | Interviews |  |  |  |
| Duration | 21 | What was the duration of the interviews or focus group? | / |  |  |  |
| Data saturation | 22 | Was data saturation discussed? | Study population |  |  |  |
| Transcripts returned | 23 | Were transcripts returned to participants for comment and/or correction? | Interviews |  |  |  |
|  | | | |  |  |  |
| *Data analysis* |  |  |  |  |  |  |
| Number of data coders | 24 | How many data coders coded the data? | Data analysis |  |  |  |
| Description of the coding tree | 25 | Did authors provide a description of the coding tree? | Results |  |  |  |
| Derivation of themes | 26 | Were themes identified in advance or derived from the data? | Data analysis |  |  |  |
| Software | 27 | What software, if applicable, was used to manage the data? | Data analysis |  |  |  |
| Participant checking | 28 | Did participants provide feedback on the findings? | Interviews |  |  |  |
| *Reporting* |  |  |  |  |  |  |
| Quotations presented | 29 | Were participant quotations presented to illustrate the themes/findings?  Was each quotation identified? e.g. participant number | Table 3 |  |  |  |
| Data and findings consistent | 30 | Was there consistency between the data presented and the findings? | Results, discussion |  |  |  |
| Clarity of major themes | 31 | Were major themes clearly presented in the findings? | Results, discussion |  |  |  |
| Clarity of minor themes | 32 | Is there a description of diverse cases or discussion of minor themes? | Results, discussion |  |  |  |

Developed from: Tong A, Sainsbury P, Craig J. Consolidated criteria for reporting qualitative research (COREQ): a 32-item checklist for interviews and focus groups. *International Journal for Quality in Health Care*. 2007. Volume 19, Number 6: pp. 349 – 357

## **Supplement 3: Topic guide**

1. **Introduction and explanation of the interview:**
2. Thanking for participation
3. Introduction of interviewer(s)
4. Explaining purpose of the interview: evaluating implementation of POM and SAM, specifically barriers and facilitators, to provide recommendations for implementation in other hospitals.
5. Explain format of interview: duration up to one hour, audio recording, data will be anonymised and used for a scientific publication. Ask permission to start audio-recorder.
6. **Can you give a short introduction about yourself?**
   1. Occupation
   2. For how long you have been employed in this hospital?
   3. Did you have any experience with implementation in this hospital prior to POM and SAM?
   4. What was your role in the implementation of POM and SAM?
7. **What was your motivation for implementing POM and SAM? [Motivation]**
8. **Can you explain how the implementation process went? [General]**
   1. What steps did the implementation process consist of?
   2. Can you reflect on those steps?
   3. Where do you stand now regarding implementation of POM and SAM?
   4. What still needs to be done?
   5. How did the ward respond to implementation of POM and SAM?
9. **What information regarding POM and SAM did you receive upon implementation? [Capability]**
   For instance,
   1. Explanation of what POM and SAM requires from patients and/or healthcare providers?
   2. Detailed description of what work processes need to be modified and examples of how this could be achieved?
   3. Study documents and/or results of a pilot study?
   4. Recommendations from colleagues who are employed in hospitals that already implemented POM and SAM?
10. **Do you feel that POM and SAM were being implemented effectively? [Capability, Opportunity]**Why or why not?
11. **How would you describe the collaborations between healthcare providers during implementation of POM and SAM? [Opportunity]**
    1. Was there a project team? If yes, who participated in the project team?
    2. How did you communicate with (other) healthcare providers in your hospital?
12. **What barriers and facilitators did you expect or experience upon implementation of POM and SAM?** **[General]** For instance, regarding:
    1. Patients
    2. Staff
    3. Finances
    4. Technological support
    5. Logistics
    6. Laws and regulations
13. **Did you feel that the resources and materials and were sufficient for effective implementation? [Opportunity]**
    1. What materials and/or resources did you perceive as helpful?
    2. What materials and/or resources were still missing / would have been needed?
14. **What could currently be done to further improve the implementation of POM and SAM? [General]**
15. **If implementation of POM and SAM could be done over, how would you approach it? [General]**
16. **Do you have any other comments on this topic? [General]**
17. **Finalizing the interview:**
    1. Summarize information.
    2. Stop audio-recorder.
    3. Thanking for participation and announcing the opportunity for member check.

| **ERIC Strategies** **Supplement : Selected ERIC Strategies based on the barriers of implementing Patient's Own Medication and Self-administration of Medication** | **Cumulative Percent** | **Trialability** | **Complexity** | **External Policy & Incentives** | **Structural Characteristics** | **Compatibility** | **Self-efficacy** | **Individual Stage of Change** | **Key Focus Area** |
| --- | --- | --- | --- | --- | --- | --- | --- | --- | --- |
| Identify and prepare champions | **185%** | 12% | 30% | 22% | 27% | 21% | 30% | 44% | Develop stakeholder interrelationships |
| Promote adaptability | **174%** | 27% | 40% | 0% | 23% | 45% | 11% | 28% | Adapt and tailor to context |
| Conduct cyclical small tests of change | **173%** | 38% | 37% | 4% | 23% | 38% | 26% | 8% | Use evaluative and iterative strategies |
| Assess for readiness and identify barriers and facilitators | **162%** | 35% | 30% | 4% | 36% | 34% | 11% | 12% | Use evaluative and iterative strategies |
| Create a learning collaborative | **149%** | 12% | 33% | 15% | 18% | 14% | 30% | 28% | Train and educate stakeholders |
| Capture and share local knowledge | **143%** | 23% | 27% | 26% | 23% | 14% | 19% | 12% | Develop stakeholder interrelationships |
| Tailor strategies | **136%** | 23% | 27% | 11% | 18% | 38% | 11% | 8% | Adapt and tailor to context |
| Model and simulate change | **124%** | 31% | 27% | 4% | 14% | 3% | 33% | 12% | Develop stakeholder interrelationships |
| Identify early adopters | **118%** | 15% | 20% | 7% | 23% | 10% | 19% | 24% | Develop stakeholder interrelationships |
| Build a coalition | **113%** | 15% | 0% | 33% | 27% | 21% | 0% | 16% | Develop stakeholder interrelationships |
| Alter incentive/allowance structures | **112%** | 0% | 7% | 41% | 18% | 10% | 4% | 32% | Utilize financial strategies |
| Conduct local consensus discussions | **112%** | 8% | 7% | 22% | 14% | 41% | 0% | 20% | Develop stakeholder interrelationships |
| Facilitation | **110%** | 23% | 20% | 4% | 9% | 24% | 22% | 8% | Provide interactive assistance |
| Provide local technical assistance | **110%** | 15% | 17% | 7% | 18% | 14% | 22% | 16% | Provide interactive assistance |
| Conduct ongoing training | **109%** | 8% | 37% | 4% | 0% | 0% | 41% | 20% | Train and educate stakeholders |
| Provide ongoing consultation | **109%** | 19% | 20% | 0% | 9% | 3% | 41% | 16% | Train and educate stakeholders |
| Stage implementation scale up | **107%** | 27% | 30% | 4% | 14% | 10% | 15% | 8% | Use evaluative and iterative strategies |
| Inform local opinion leaders | **107%** | 23% | 13% | 22% | 14% | 3% | 4% | 28% | Develop stakeholder interrelationships |
| Develop a formal implementation blueprint | **107%** | 19% | 43% | 7% | 18% | 3% | 11% | 4% | Use evaluative and iterative strategies |
| Make training dynamic | **99%** | 0% | 10% | 0% | 5% | 3% | 41% | 40% | Train and educate stakeholders |
| Conduct educational meetings | **86%** | 8% | 13% | 15% | 5% | 10% | 15% | 20% | Train and educate stakeholders |
| Organize clinician implementation team meetings | **78%** | 8% | 20% | 0% | 14% | 14% | 11% | 12% | Develop stakeholder interrelationships |
| Purposely reexamine the implementation | **75%** | 15% | 17% | 11% | 0% | 28% | 0% | 4% | Use evaluative and iterative strategies |
| Conduct local needs assessment | **69%** | 19% | 3% | 7% | 18% | 21% | 0% | 0% | Use evaluative and iterative strategies |
| Fund and contract for clinical innovation | **66%** | 8% | 3% | 15% | 14% | 10% | 4% | 12% | Utilize financial strategies |
| Involve executive boards | **66%** | 8% | 0% | 41% | 14% | 3% | 0% | 0% | Develop stakeholder interrelationships |
| Use an implementation adviser | **63%** | 23% | 10% | 4% | 5% | 10% | 7% | 4% | Develop stakeholder interrelationships |
| Conduct educational outreach visits | **61%** | 8% | 7% | 0% | 0% | 0% | 22% | 24% | Train and educate stakeholders |
| Audit and provide feedback | **60%** | 15% | 3% | 0% | 5% | 7% | 22% | 8% | Use evaluative and iterative strategies |
| Visit other sites | **60%** | 12% | 3% | 7% | 5% | 10% | 15% | 8% | Develop stakeholder interrelationships |
| Shadow other experts | **60%** | 4% | 7% | 0% | 5% | 3% | 33% | 8% | Train and educate stakeholders |
| Develop educational materials | **59%** | 0% | 13% | 4% | 0% | 3% | 19% | 20% | Train and educate stakeholders |
| Mandate change | 49% | 4% | 7% | 15% | 5% | 3% | 4% | 12% | Change infrastructure |
| Develop and implement tools for quality monitoring | 49% | 12% | 7% | 11% | 5% | 3% | 4% | 8% | Use evaluative and iterative strategies |
| Develop and organize quality monitoring systems | 48% | 8% | 10% | 15% | 5% | 3% | 7% | 0% | Use evaluative and iterative strategies |
| Involve patients/consumers and family members | 46% | 0% | 0% | 11% | 9% | 10% | 4% | 12% | Engage consumers |
| Change physical structure and equipment | 42% | 0% | 3% | 0% | 32% | 7% | 0% | 0% | Change infrastructure |
| Use advisory boards and workgroups | 42% | 8% | 0% | 15% | 5% | 3% | 7% | 4% | Develop stakeholder interrelationships |
| Promote network weaving | 42% | 0% | 0% | 11% | 23% | 0% | 4% | 4% | Develop stakeholder interrelationships |
| Centralize technical assistance | 40% | 4% | 10% | 0% | 5% | 10% | 11% | 0% | Provide interactive assistance |
| Develop academic partnerships | 39% | 4% | 0% | 11% | 5% | 0% | 7% | 12% | Develop stakeholder interrelationships |
| Use train the trainer strategies | 37% | 4% | 7% | 4% | 0% | 0% | 15% | 8% | Train and educate stakeholders |
| Provide clinical supervision | 36% | 0% | 7% | 0% | 0% | 10% | 11% | 8% | Provide interactive assistance |
| Revise professional roles | 36% | 0% | 3% | 4% | 18% | 10% | 0% | 0% | Support clinicians |
| Recruit, designate and train for leadership | 33% | 0% | 7% | 0% | 18% | 0% | 4% | 4% | Develop stakeholder interrelationships |
| Create or change credentialing and/or licensure standards | 31% | 0% | 0% | 19% | 5% | 0% | 0% | 8% | Change infrastructure |
| Obtain and use patients/consumers and family feedback | 30% | 4% | 0% | 0% | 5% | 10% | 4% | 8% | Use evaluative and iterative strategies |
| Place innovation on fee for service lists/formularies | 30% | 0% | 0% | 19% | 0% | 3% | 0% | 8% | Utilize financial strategies |
| Facilitate relay of clinical data to providers | 30% | 4% | 3% | 4% | 0% | 3% | 7% | 8% | Support clinicians |
| Obtain formal commitments | 28% | 4% | 0% | 15% | 9% | 0% | 0% | 0% | Develop stakeholder interrelationships |
| Create new clinical teams | 27% | 0% | 3% | 0% | 9% | 7% | 7% | 0% | Support clinicians |
| Distribute educational materials | 27% | 12% | 3% | 0% | 0% | 0% | 4% | 8% | Train and educate stakeholders |
| Change liability laws | 25% | 0% | 0% | 19% | 0% | 7% | 0% | 0% | Change infrastructure |
| Change accreditation or membership requirements | 23% | 0% | 0% | 15% | 5% | 0% | 0% | 4% | Change infrastructure |
| Access new funding | 23% | 4% | 3% | 7% | 5% | 3% | 0% | 0% | Utilize financial strategies |
| Use data experts | 22% | 12% | 3% | 4% | 0% | 0% | 4% | 0% | Adapt and tailor to context |
| Change service sites | 21% | 0% | 0% | 4% | 14% | 3% | 0% | 0% | **Change infrastructure** |

Level-1 strategies: ≥50% expert agreement on effectiveness in addressing a specific CFIR construct;
Level-2 strategies: 20-50% expert agreement on effectiveness in addressing a specific CFIR construct.
